# Supplementary material for: Autumn freeze-thaw events carry over to depress late-winter reproductive performance in Canada jays
Source: R Soc Open Sci. 2019 Apr 10;6(4):181754. doi: 10.1098/rsos.181754 (PMC6502392; doi:10.1098/rsos.181754)
Supplement: Table S1 [file rsos181754supp1.docx]

**Table S1**

| **Food Item** | **Food Group** | **Freezing Point (°C)** | **Reference** |
| --- | --- | --- | --- |
| Reindeer meat | Meat | -3.1 | Roos (1986) |
| Venison | Meat | -0.74 | Dickerson (1968) |
| Chicken | Meat | -0.79 | Dickerson (1968) |
| Poultry | Meat | -2.80 | ASHRAE (1967) |
| Turkey | Meat | -2.80 | Murakami and Okos (1989) |
| Lamb – Leg | Meat | -1.47 | Mellor (1983) |
| Veal | Meat | -0.74 | Dickerson (1968) |
| Veal | Meat | -1.75 | Murakami and Okos (1989) |
| Veal (calf) | Meat | -0.68 | Pham (1987) |
| Pork –Muscle | Meat | -1.75 | Murakami and Okos (1989) |
| Pork –Muscle | Meat | -1.00 | Murakami and Okos (1989) |
| Pork –Muscle | Meat | -0.90 | Levy (1979) |
| Pork –Muscle | Meat | -2.20 | ASHRAE (1967) |
| Pork –Shoulder | Meat | -2.20 | ASHRAE (1967) |
| Beef –Carcass | Meat | -2.20 | ASHRAE (1967) |
| Beef –Fat | Meat | -2.20 | ASHRAE (1967) |
| Beef –Flank | Meat | -1.75 | Murakami and Okos (1989) |
| Beef –Liver | Meat | -1.70 | ASHRAE (1967) |
| Beef –Muscle | Meat | -13.46 | Pham (1987) |
| Beef –Muscle | Meat | -4.09 | Succar and Hayakawa (1983) |
| Beef –Muscle | Meat | -2.80 | Chen (1986) |
| Beef –Muscle | Meat | -3.00 | Levy (1979) |
| Beef –Muscle | Meat | -3.63 | Pham (1987) |
| Beef –Muscle | Meat | -2.02 | Succar and Hayakawa (1983) |
| Beef –Muscle | Meat | -2.00 | Levy (1979) |
| Beef –Muscle | Meat | -1.20 | Mannapperuma and Singh (1989) |
| Beef –Muscle | Meat | -1.76 | Succar and Hayakawa (1983) |
| Beef –Muscle | Meat | -1.00 | Mannapperuma and Singh (1989) |
| Beef –Muscle | Meat | -1.01 | Succar and Hayakawa (1983) |
| Beef –Muscle | Meat | -1.10 | Mascheroni and Calvelo (1980) |
| Beef –Muscle | Meat | -0.75 | Murakami and Okos (1989) |
| Beef –Muscle | Meat | -0.99 | Succar and Hayakawa (1983) |
| Beef –Muscle | Meat | -0.99 | Pham (1987) |
| Beef –Muscle | Meat | -1.00 | Chen (1986) |
| Beef –Muscle | Meat | -0.63 | Dickerson (1968) |
| Beef –Muscle | Meat | -1.77 | Heldman (1974) |
| Beef –Muscle | Meat | -0.82 | Sheard et al. (1990) |
| Beef –Muscle | Meat | -1.11 | Mellor (1983) |
| Beef –Muscle | Meat | -0.73 | Pham (1987) |
| Beef –Muscle | Meat | -0.80 | Levy (1979) |
| Bilberry | Berry | -1.10 | Heldman and Singh (1981) |
| Blackberry | Berry | -1.40 | ASHRAE (1967) |
| Blueberry | Berry | -1.90 | ASHRAE (1967) |
| Cranberry | Berry | -2.60 | ASHRAE (1967) |
| Raspberry | Berry | -1.10 | ASHRAE (1967) |
| Raspberry | Berry | -1.22 | Heldman and Singh (1981) |
| Dewberry | Berry | -1.60 | ASHRAE (1967) |
| *Agaricus bisporus* | Mushroom | -1.5 | Guizani et al. 2013 |
| Mushroom | Mushroom | -1.6 | Haiying et al. 2007 |
| Mushroom | Mushroom | -1.3 | Haiying et al. 2007 |
| Mushroom | Mushroom | -0.7 | Haiying et al. 2007 |
