# Supplementary material for: Autumn freeze-thaw events carry over to depress late-winter reproductive performance in Canada jays
Source: R Soc Open Sci. 2019 Apr 10;6(4):181754. doi: 10.1098/rsos.181754 (PMC6502392; doi:10.1098/rsos.181754)
Supplement: Table S2 [file rsos181754supp2.docx]

**Table S2**

| **Food Group** | **Mean** | **SE** | **Range** | **N** |
| --- | --- | --- | --- | --- |
| Meat | -1.936 | 0.5051 | -13.46 to -0.63 | 40 |
| Berry | -1.560 | 0.7317 | -2.60 to -1.10 | 7 |
| Mushroom | -1.275 | 0.2016 | -1.6 to -0.7 | 4 |
