# Supplementary material for: Autumn freeze-thaw events carry over to depress late-winter reproductive performance in Canada jays
Source: R Soc Open Sci. 2019 Apr 10;6(4):181754. doi: 10.1098/rsos.181754 (PMC6502392; doi:10.1098/rsos.181754)
Supplement: Table S3 [file rsos181754supp3.docx]

**Table S3.**

| **Model** | **Freeze-Fall** | **Temp-Pre** | **Female Age** | **Lay Date** |
| --- | --- | --- | --- | --- |
| Freeze-Fall + Temp-Pre + Freeze-Fall*Lay Date | -0.081  (-0.15 - -0.001) | -0.068  (-0.13 – 0.008) | 0.064  (-0.001 – 0.12) | -0.234  (-0.31 - -0.17) |
| Freeze-Fall + Temp-Pre | -0.071  (-0.14 - 0.006) | -0.068  (-1.35 – 0.008) | 0.065  (6.3e^-5^ – 0.12) | -0.236  (-0.31 - -0.17) |
| Freeze-Fall + Temp-Pre + Freeze-Pre | -0.073  (-0.14 – 0.004) | -0.100  (-0.20 – 0.016) | 0.065  (2.0e^-4^ – 0.12) | -0.238  (-0.31 - -0.17) |
| Freeze-Fall + Temp-Fall + Temp-Pre | -0.064  (-0.068 – 0.039) | -0.064  (-0.13 – 0.014) | 0.065  (-1.7e^-4^ – 0.12) | -0.238  (-0.31 - -0.17) |

Freeze = frequency of freeze-thaw events, Temp = mean temperature, Fall = fall caching period (October – November), Pre = Pre-breeding period (January – February)
